# On Gleet and Its Treatment

**Published:** 1850-07

**Authors:** 


*On Gleet and its Treatment.*—Christopher Johnson, M. D., of Baltimore recommends the following treatment for gleet:

℞. Strychnia, grs. ij.;
Acid. Nit. fort., gtts. iv.;
Aqua, ℥ij.

Ft. sol. M. Signa—inject one drachm, thrice a day, after urination.

℞. Ext. nucis vomica, grs. xij.;
Sulph. quinia,
Ext. hyosciami, ä. grs. xxiv.

M. In pil. No. xxiv. divid. Signa—two pills to be taken one hour before each meal.

Dr. Johnson recommends also the use of lean meats, and abstinance for a fortnight from salted and smoked meats, and from saccharine articles of diet in the usual proportion.

